# Supplementary material for: Physicochemical and Biological Effects on Activated Sludge Performance and Activity Recovery of Damaged Sludge by Exposure to CeO2 Nanoparticles in Sequencing Batch Reactors
Source: Int J Environ Res Public Health. 2019 Oct 21;16(20):4029. doi: 10.3390/ijerph16204029 (PMC6843984; doi:10.3390/ijerph16204029)
Supplement: Supplementary file 1 [file ijerph-16-04029-s001.pdf]

Supplementary material

Towards physicochemical and biological effects on activated sludge performance and activity recovery of damaged sludge by exposing to CeO_2 nanoparticles in sequencing batch reactors

Qian Feng[1, 2*], Yaqing Sun[2], Yang Wu[2], Zhaoxia Xue[1, 2], Jingyang Luo[1, 2], Fang Fang[1, 2], Chao Li[1, 2], Jiashun Cao[1, 2]

[1] Key Laboratory of Integrated Regulation and Resource Development on Shallow Lakes, Ministry of Education, Hohai University, Nanjing 210098, China

[2] College of Environment, Hohai University, Nanjing 210098, China

* **Corresponding author**:

Dr. Qian Feng, E-mail: xiaofq@hhu.edu.cn

Figure Captions

Figure S1. The magnified SEM images of the activated sludge from R4. (The yellow circles in Figure S1 a, b, c, d represent the filamentous bacteria, spherical bacteria, rod-shaped bacteria and the attached CeO_2 NPs, respectively.).

Figure S2. Relative LDH release during one cycle after 1-d and 10-week exposure to CeO_2 NPs at different concentrations, respectively. Error bars represent standard deviations of triplicate measurement.

Figure S3. The energy spectrum of activated sludge within 2-week recovery time relieved from the 10-week exposure to 0.1 (a), 1 (b), and 10 (c) mg/L CeO_2 NPs.

Figure S4. Heat-map of the four reactors within 2-week recovery time relieved from the 10-week exposure to different CeO_2 NPs concentration.

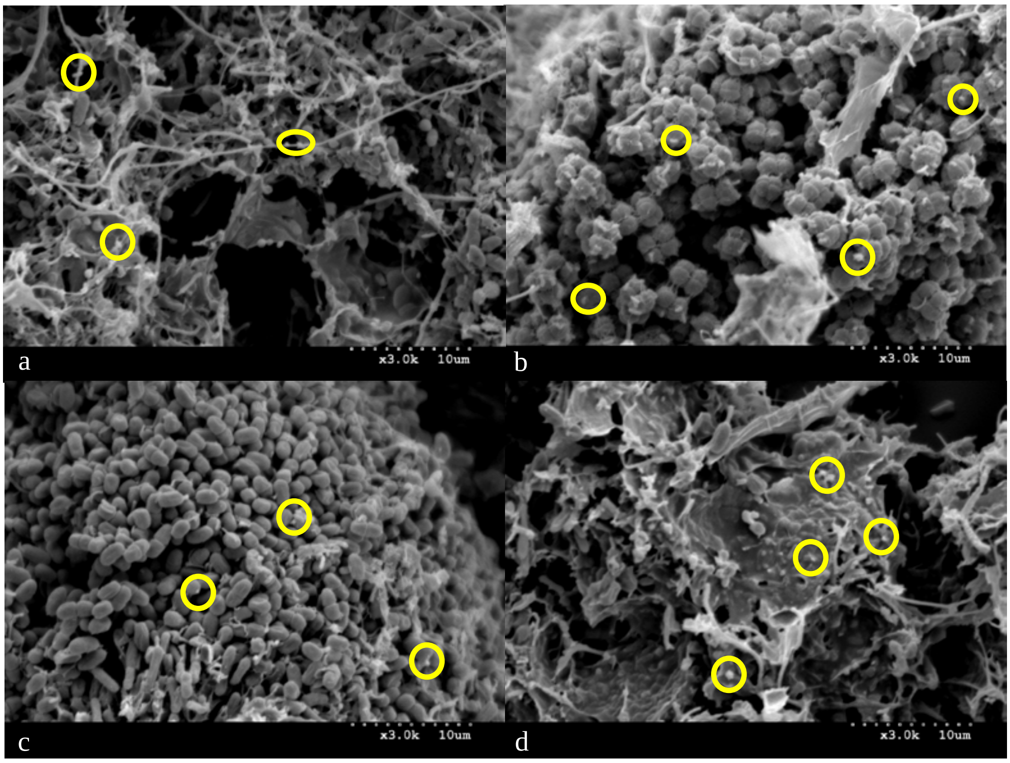

Figure S1. The magnified SEM images of the activated sludge from R4. (The yellow circles in Figure S1 a, b, c, d represent the filamentous bacteria, spherical bacteria, rod-shaped bacteria and the attached CeO_2 NPs, respectively.).

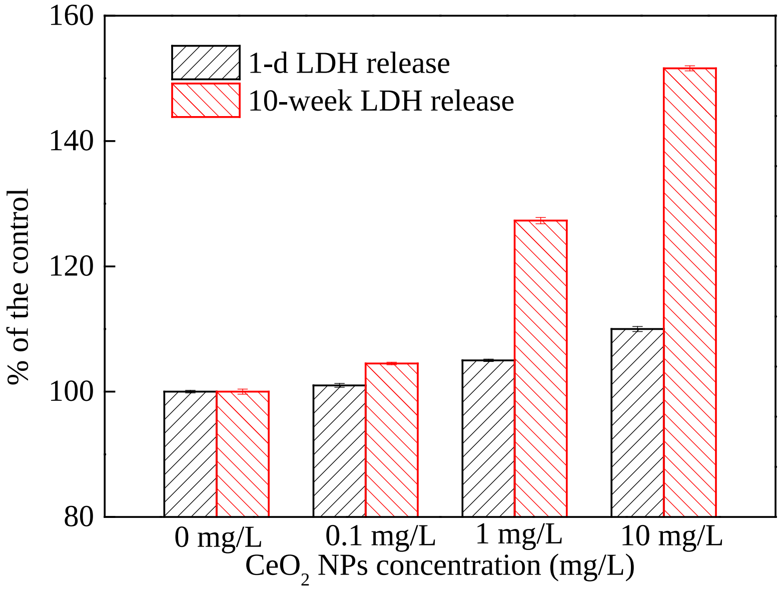

Figure S2. Relative LDH release during one cycle after 1-d and 10-week exposure to CeO_2 NPs at different concentrations, respectively. Error bars represent standard deviations of triplicate measurement.

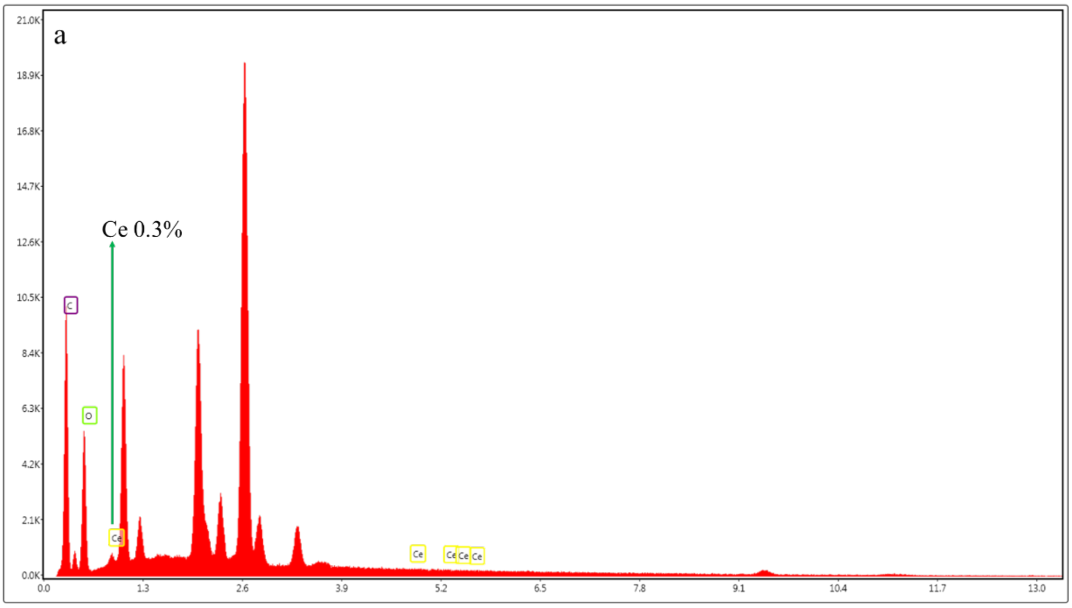
a
Ce 0.3%
C
O
Ce
Ce
Ce
Ce
Ce
21.0K
18.9K
16.8K
14.7K
12.6K
10.5K
8.4K
6.3K
4.2K
2.1K
0.0K
0.0
1.3
2.6
3.9
5.2
6.5
7.8
9.1
10.4
11.7
13.0

(a)

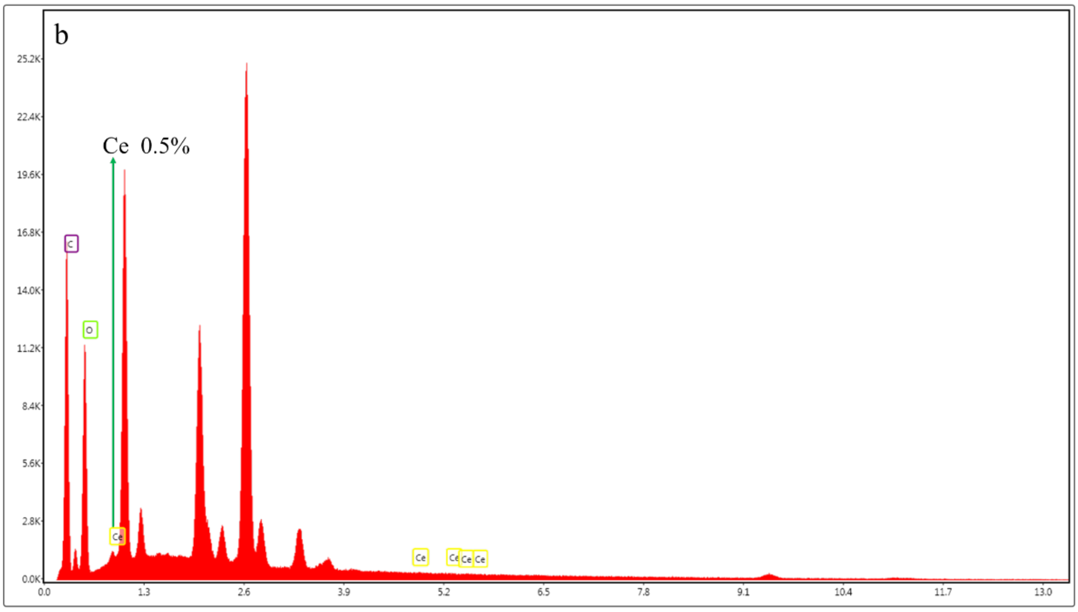
b
Ce 0.5%
C
O
Ce
Ce
Ce
Ce
Ce
25.2K
22.4K
19.6K
16.8K
14.0K
11.2K
8.4K
5.6K
2.8K
0.0K
0.0
1.3
2.6
3.9
5.2
6.5
7.8
9.1
10.4
11.7
13.0

(b)

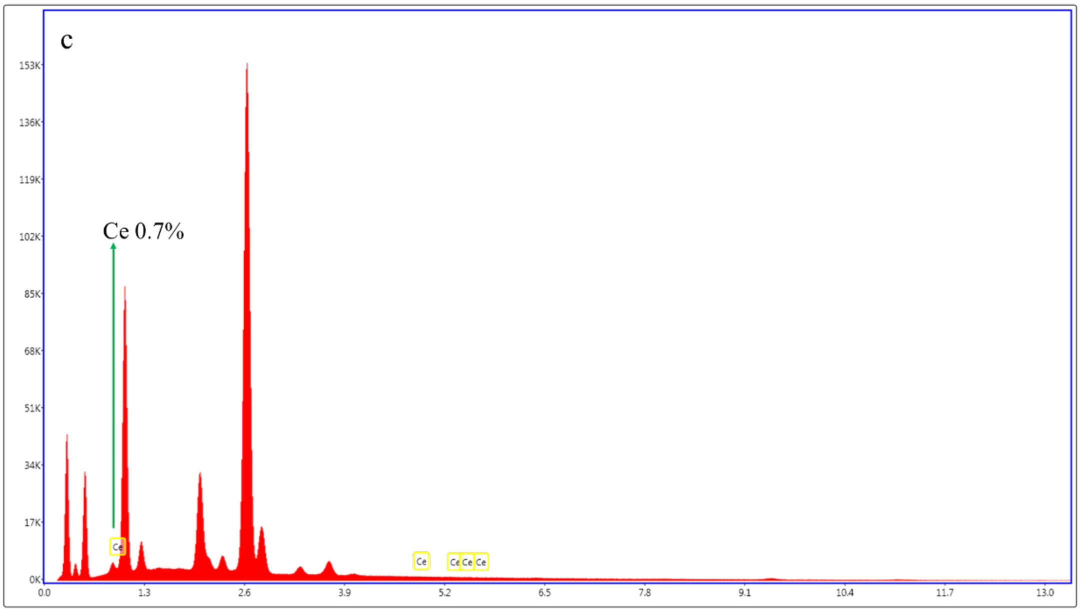

(c)

Figure S3. The energy spectrum of activated sludge within 2-week recovery time relieved from the 10-week exposure to 0.1 (a), 1 (b), and 10 (c) mg/L CeO_2 NPs.

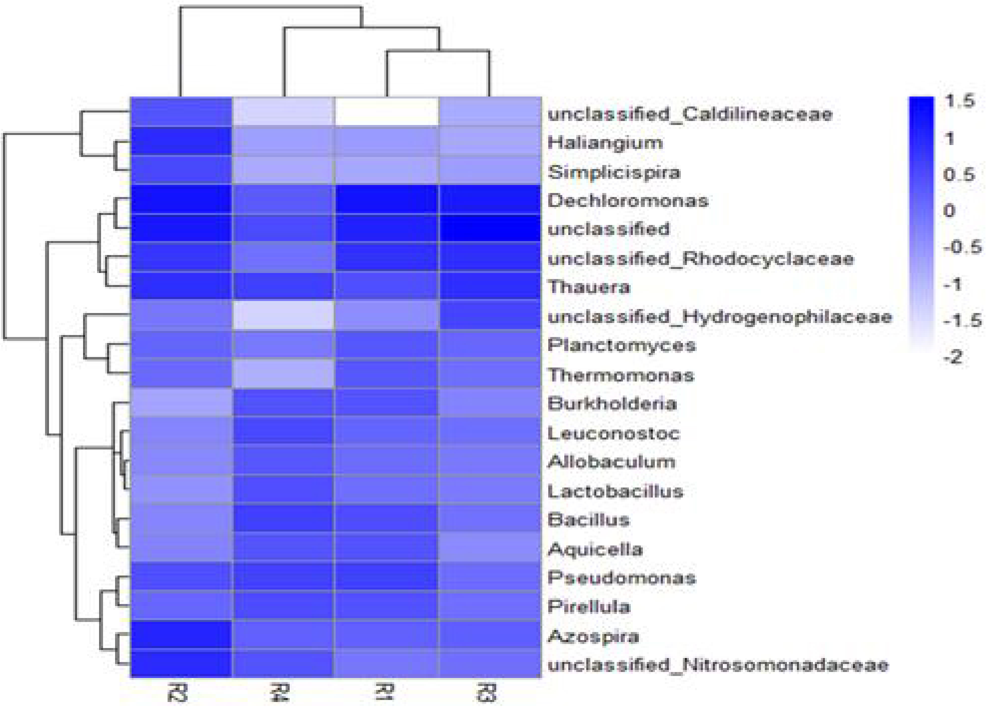

Figure S4. Heat-map of the four reactors within 2-week recovery time relieved from the 10-week exposure to different CeO_2 NPs concentration.
